# Unusual presentation of primary hyperparathyroidism: report of three cases

**DOI:** 10.1186/s12880-015-0064-1

**Published:** 2015-07-16

**Authors:** Ruibin Huang, Ruyao Zhuang, Yuan Liu, Tianti Li, Jiexiong Huang

**Affiliations:** Department of Radiology, The First Affiliated Hospital of the Medical College of Shantou University, Shantou, China; Department of Pathology, The First Affiliated Hospital of the Medical College of Shantou University, Shantou, China

**Keywords:** Primary hyperparathyroidism, Brown tumor, Osteitis fibrosa cystica, Giant cell tumor

## Abstract

**Background:**

Primary hyperparathyroidism is an endocrinopathic condition characterized by hypersecretion of parathyroid hormone. Excess parathyroid hormone results in an altered state of osseous metabolism involving bone resorption and tissue change known as osteitis fibrosa cystica, which is the end stage of primary hyperparathyroidism. Osteitis fibrosa cystica is associated with the development of brown tumors, which are rare because hyperparathyroidism is now usually diagnosed and treated before symptoms develop. Brown tumors are rarely the first symptom of hyperparathyroidism and can occasionally be mistaken for malignancy.

**Case Presentation:**

We herein report three cases of primary hyperparathyroidism with an unusual presentation of brown tumors. All three patients were Asian. In the first case, a 42-year-old man was admitted with a mass mimicking a malignant bone neoplasm in the right mandible as the first manifestation of primary hyperparathyroidism. The second case involved a 25-year-old man admitted with a fracture of his right femur. The third case involved a 43-year-old man with multiple brown tumors in both lower limbs. All three patients underwent successful parathyroidectomy for parathyroid adenomas; one case was complicated by a papillary thyroid carcinoma.

**Conclusion:**

Complete evaluation of the medical history and biochemical and radiographic findings is necessary to achieve a correct diagnosis and avoid unnecessary bone resections in patients with primary hyperparathyroidism.

## Background

The parathyroid glands secrete parathyroid hormone (PTH), which is involved in regulating the metabolism of calcium and phosphorus. PTH plays an important role in tooth development and bone mineralization and increases bone resorption. Excessive secretion of PTH is termed hyperparathyroidism (HPT), which is an endocrine condition categorized as primary, secondary, or tertiary. Primary HPT (PHPT) is an endocrinopathic condition characterized by hypersecretion of PTH, which may be caused by an adenoma (solitary or multiple), idiopathic hyperplasia, or a parathyroid carcinoma. Secondary HPT is caused by hypocalcaemia or vitamin D deficiency acting as a stimulus of excessive PTH production. Chronic renal failure is the main cause of secondary HPT. Tertiary HPT is caused by the development of autonomous parathyroid hyperplasia after long-standing secondary HPT, most often in patients with renal failure [1]. Most cases of PHPT (80 %–85 %) are caused by a solitary adenoma, 15 % to 20 % are due to parathyroid gland hyperplasia, and <0.5 % are caused by parathyroid carcinoma [2, 3]. Renal calculi have been reported in 10 % to 25 % of cases, and the frequency of bone disease among patients with PHPT is 10 % to 20 % [4].

Skeletal involvement in classic PHPT is characterized by a strikingly high rate of osteoclastic bone resorption and is accompanied by a cellular repair process that results in the accumulation of fibrous stroma and connective tissue cells along with multinucleated giant cells. Thus, brown tumors have been described as resulting from an imbalance between osteoclastic and osteoblastic activity, resultant resorption with fibrous replacement of the bone, and eventual osteitis fibrosa cystica (OFC).

Brown tumors are reactive lesions and do not represent true neoplasms. They may be difficult to diagnose because they present clinically and radiologically as other diseases such as giant cell tumors, multiple bone metastases, or multiple myeloma [5, 6]. Moreover, from a histological perspective, differential diagnosis among brown tumors, giant cell granulomas, and giant cell bone tumors may be very difficult without an accurate and complete preoperative clinical evaluation.

Brown tumors involving the jaw bones as the first manifestation of PHPT and pathological fractures that lead to a diagnosis of PHPT are not commonly described [7–11]. We herein report three Asian patients with PHPT with an unusual first manifestation of brown tumors.

### Case presentations

#### Case 1

**Fig. 1:**
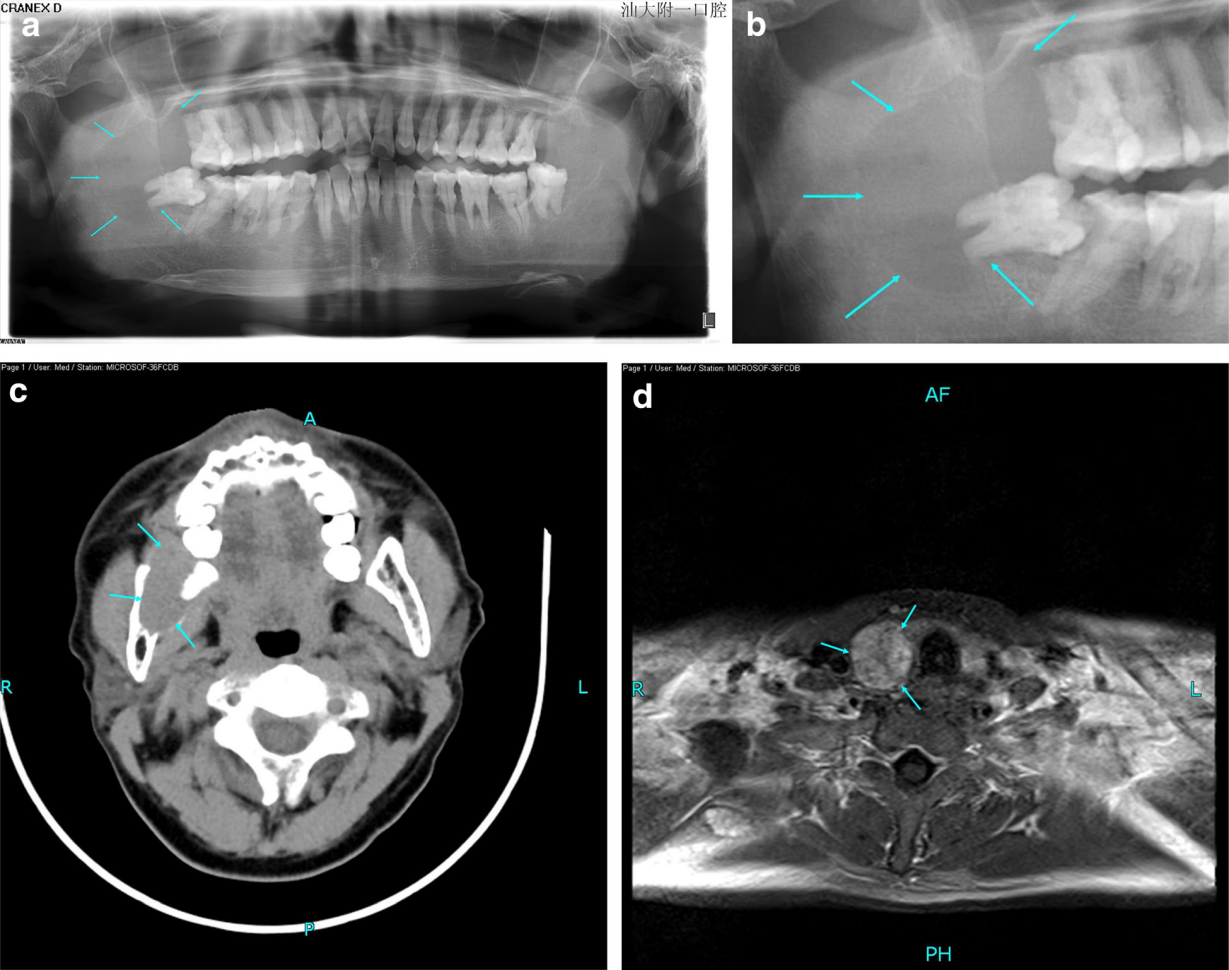
(**a**, **b**) Panoramic radiograph revealing a well-defined osteolytic lesion in the right mandible. (**c**) Axial CT scan showing an expansionary cystic bone lesion of the right mandible, penetrating the cortex and forming a peripheral soft tissue mass. (**d**) Enhanced MRI of the neck showing a 2.5-cm enhancing nodular mass on the right parathyroid

A 42-year-old man with no known disease history noticed a mass on the right side of his face, which gradually generalized. One month later, he presented to our hospital for an examination. He had no other associated complaints or any significant family history. Intraoral examination revealed a reddish, nontender, hard, ill-defined sessile swelling in the right side of the mandible. Panoramic radiography revealed a well-defined, osteolytic lesion measuring 40 × 30 mm in the right mandible (Fig. 1a, b). Computed tomography (CT) revealed an expansionary cystic bone lesion that penetrated the cortex, forming a peripheral soft tissue mass (Fig. 1c). The bone mass was suspected to be a malignant neoplastic lesion. ^m99^Tc bone scintigraphy revealed multiple hypermetabolic foci in the right acromion, left maxilla, and bilateral mandibular bone. Urinary tract ultrasonography revealed multiple nephroliths on the left side and mild hydronephrosis caused by lower ureteral calculi on the right side. Biopsy of the right mandibular mass revealed spindle-shaped mesenchymal cells with a large number of multinucleated giant cells throughout the lesion. The benign lesion was identified as a giant cell granuloma. The mandibular lesion was removed before performing magnetic resonance imaging (MRI) of the thyroid gland and neck.

Laboratory data are shown in Table 1 and included a PTH level of 939.8 pg/ml (reference range, 15–88 pg/ml), serum calcium level of 2.93 mmol/L (reference range, 2.08–2.80 mmol/L), and alkaline phosphatase (ALP) level of 422 U/L (reference range, 45–125 U/L) before parathyroidectomy. Urine testing was positive for calcium oxalate crystals. Glucose, bilirubin, uric acid, protein, albumin, and globulin levels were all normal. Serum immunoelectrophoresis findings were also normal. The concentrations of IgG, IgA, IgM, C3, C4, and C-reactive protein were within the normal range.

**Table 1:** Laboratory data before and after parathyroidectomy in the three patientsPTH, parathyroid hormone; ALP, alkaline phosphatase; Ca, calcium; −, not available

	Reference range, adults	Admission	Before parathyroidectomy	After parathyroidectomy
PTH level (pg/ml)	15–88	Case 1: −	Case 1: 939.80	Case 1: 66.40
		Case 2: −	Case 2: 1399.80	Case 2: 65.40
		Case 3: −	Case 3: 1084.45	Case 3: 80.20
Ca level (mmol/L)	2.08–2.80	Case 1: 3.18	Case 1: 2.93	Case 1: 2.13
		Case 2: 3.59	Case 2: 3.37	Case 2: 2.56
		Case 3: 2.91	Case 3: 3.53	Case 3: 2.24
Phosphorus level (mmol/L)	0.80–1.50	Case 1: −	Case 1: −	Case 1: 0.75
		Case 2: 0.78	Case 2: −	Case 2: 0.52
		Case 3: −	Case 3: −	Case 3: −
ALP (U/L)	45–125	Case 1: 422	Case 1: −	Case 1: −
		Case 2: 1367	Case 2: −	Case 2: −
		Case 3: 1154	Case 3: 387	Case 3: −
Creatine (μmol/L)	45–133	Case 1: 131	Case 1: −	Case 1: −
		Case 2: 94	Case 2: 108	Case 2: 217
		Case 3: 51	Case 3: 60	Case 3: 66

These findings suggested that PHPT initially manifested as a brown tumor of the jaw. Contrast MRI of the neck confirmed the existence of a 2.5-cm nodular mass on the lower right parathyroid (Fig. 1d). Mini-invasive parathyroidectomy was performed. Histopathology confirmed that the resected lesion was a parathyroid adenoma.

The patient recovered well postoperatively, and his serum calcium (2.13 mmol/L) and PTH (66.40 pg/ml) levels normalized within 1 week. Detailed biochemical data observed before and after parathyroidectomy are shown in Table 1. The patient was lost to long-term follow-up after clinical improvement.

#### Case 2

**Fig. 2:**
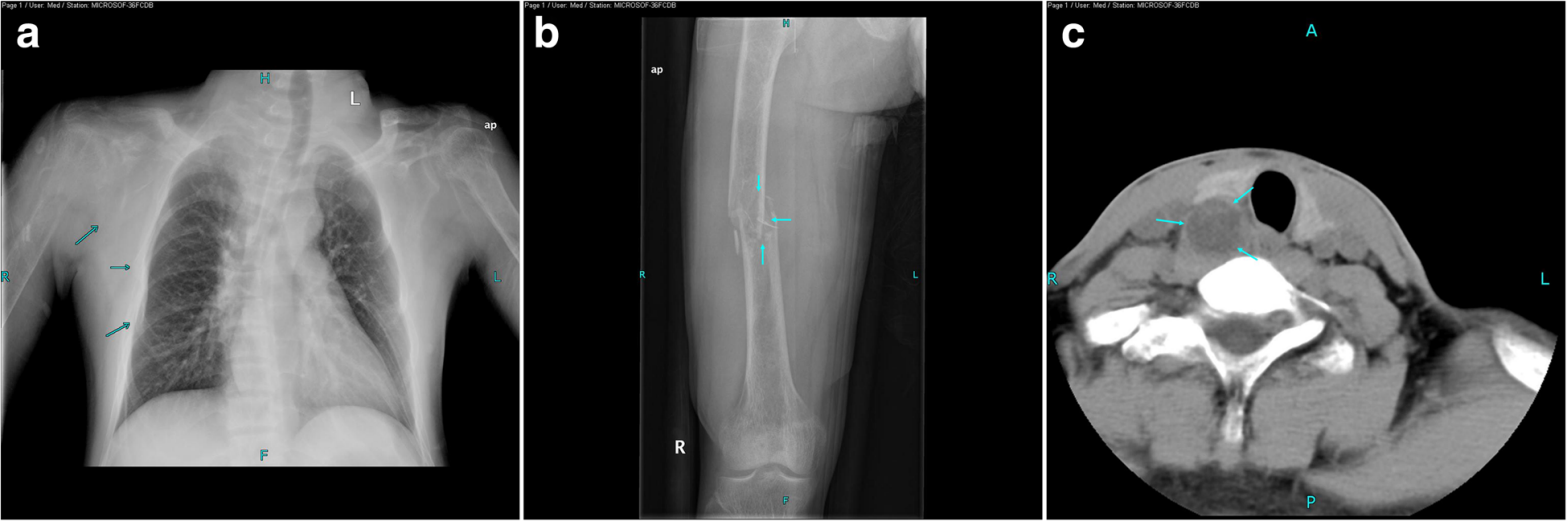
(**b**) Radiographs of a pathological fracture of the right femur. (**a**) Chest radiograph showing extensive osteolysis of the thoracic bones and several old fractures on the right scapula and ribs (arrow). (**c**) CT scan of the neck (axial view) showing a 3.5 × 2.5 × 2.0cm nodular mass on the right parathyroid

A 25-year-old man developed swelling and pain of the right shoulder after tripping on a step and falling onto his right side. Two weeks later, he was hospitalized with swelling and deformity of the right thigh when crossing the legs. Radiography confirmed a comminuted fracture of the femur secondary to a lytic bone lesion (Fig. 2b). Chest radiography showed extensive osteolysis of the thoracic bones and several old fractures (Fig. 2a). His calcium level was 3.59 mmol/L (reference range, 2.08–2.80 mmol/L) and creatinine level was 94 μmol/L (reference range, 45–133 μmol/L). Serum immunoelectrophoresis findings were normal. The levels of IgG, IgA, IgM, alpha-fetoprotein, and carcinoembryonic antigen 2 were normal. The circulating level of PTH was 1399.80 pg/ml (reference range, 15–88 pg/ml), and the diagnosis of PHPT was confirmed. CT of the neck revealed a nodular mass measuring 3.5 × 2.5 × 2.0 cm in the lower right parathyroid gland (Fig. 2c).

Parathyroidectomy was performed, and the lesion was histologically confirmed to be a benign parathyroid adenoma. The patient recovered well postoperatively, and his serum calcium (2.08 mmol/L) and PTH (65.40 pg/ml) levels normalized within 2 weeks. The patient’s biochemical data before and after parathyroidectomy are shown in Table 1. Surgical intervention for the pathologic fracture was contraindicated because of serious osteoporosis. Thus, conservative treatment involving fracture reduction and splint fixation was performed. This patient was still being followed up at the time of this writing.

#### Case 3

**Fig. 3:**
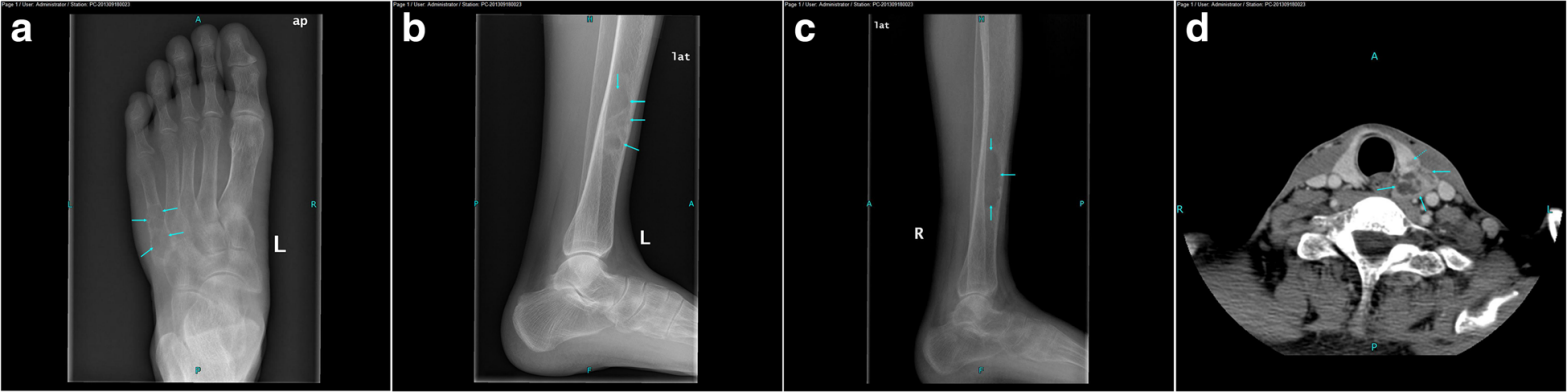
(**a**-**c**) Radiographs showing multiple mild but expansive osteolytic lesions in the bilateral distal tibia and left fifth metatarsal bone. (**d**) Axial CT scan of the neck showing a mass lesion measuring 2.7 × 1.4 cm on the left parathyroid (arrow) and a round lesion measuring 1.5 × 1.0 cm in the left thyroid lobe (dotted arrow)

A 43-year-old man presented with a 1-month history of left foot swelling with no other associated complaints or significant medical history. The swelling was mildly tender in the left fifth metatarsal bone with no reddish color or throbbing sensation. Radiography revealed multiple mild but expansive lytic bone lesions in the bilateral distal tibia and left fifth metatarsal bone (Fig. 3a–c). Biopsy of the left fifth metatarsal bone and left distal tibia lesions revealed numerous osteoclast-like giant plurinuclear cells without necrosis, mitoses, or histological signs of malignancy; these findings were compatible with a giant cell tumor of the bone. The patient underwent excision of the left fifth metatarsal bone and curettage of the lesion in the left distal tibia. Three weeks after surgery, the first medical and laboratory follow-up examinations revealed a serum calcium level of 2.91 mmol/L (reference range, 2.08–2.80 mmol/L), ALP level of 1154 U/L (reference range, 45–132 U/L), and PTH level of 1084.45 pg/ml (reference range, 15–88 pg/ml). A review of the patient’s medical records revealed hypercalcemia, which had been underestimated at the first examination. The patient had no family history of parathyroid disease. The creatinine level was 51 μmol/L (reference range, 45–133 μmol/L).

PHPT was suspected. Enhancement CT of the neck revealed a round mass measuring 2.7 × 1.4 cm behind and below the left thyroid lobe and a round lesion measuring 1.5 × 1.0 cm located in the left thyroid lobe (Fig. 3d). Parathyroidectomy was performed. Histological examination confirmed the diagnosis of adenoma of the left parathyroid gland and papillary thyroid carcinoma of the left thyroid lobe. Biochemical data observed before and after parathyroidectomy are shown in Table 1. After a review of bone slices, the final diagnosis was a brown tumor probably due to long-standing underestimated PHPT.

## Conclusions

The incidence of PHPT is difficult to assess and may vary widely from 0.4 to 21.6 cases per 100,000 person-years [12, 13]. Additionally, racial or regional differences in the incidence and prevalence of PHPT may exist [14]. The incidence of PHPT is nearly two or three times more common in women than in men [6, 14, 15]. In most patients with PHPT (80 %–85 %), the disease develops due to a parathyroid adenoma, in 15 % to 20 % due to parathyroid gland hyperplasia, and in <0.5 % due to parathyroid carcinoma [2, 3]. Before the 1970s, PHPT was a disease caused by recurrent kidney stones, brown tumors, neuromuscular dysfunction, and symptomatic hypercalcemia [16, 17]; today, it can be diagnosed in the early and asymptomatic period because of advances in blood analysis and a growing awareness of this disease [18–21]. However, normocalcemic HPT may be an early form of the disease [8, 22].

Although many systems are affected in patients with PHPT, the most pronounced alternations are observed in the bone. Classic skeletal lesions are bone resorption, OFC, and brown tumors. With the recent development of imaging and laboratory screening methods, however, hypercalcemia due to primary or secondary HPT can often be detected early, with a decrease in frequency of OFC and brown tumors. A brown tumor is the localized form of OFC [23]. It is more common in people aged >50 than <50 years and three times more common in women than in men; the preferential locations are the facial skeleton (particularly the mandible) [20] and the ends of long bones and ribs [24]. The incidence of brown tumors in patients with PHPT is 1.5 % to 1.7 % [25], and these tumors rarely present as the first manifestation of PHPT, as in our cases.

The term “brown tumor” is derived from the characteristic appearance of a brown-colored material within the cystic lesion, which is caused by high vascularity, hemorrhage, and hemosiderin deposits [26]. Brown tumors are not true neoplasms, but they can be locally aggressive and mimic malignancies. In some cases, they may lead primarily to the differential diagnosis of other giant cell lesions (reparative giant cell granuloma, cherubism, aneurysmal bone cyst, or true giant cell tumor) or a metastatic lesion because of the rarity of multiple lesions of this type [5, 27, 28]. This was demonstrated in our first patient, in whom medical imaging findings suggested a malignant bone neoplasm.

On radiography and CT, brown tumors are seen as lytic lesions or sclerotic lesions with regular borders and no cortical disruption, periosteal reaction, or inflammatory signs [29]. MRI shows variable intensities on T2-weighted images and intense enhancement on T1-weighted contrast images [9, 30]. The appearance on radiographs and CT is variable and not always specific and can mimic a malignant tumor [4, 5, 31]. In our first patient, the expansionary cystic bone lesion in the right mandible penetrated the cortex and formed a peripheral soft tissue mass, mimicking a malignant neoplastic lesion and thus confusing clinicians regarding the correct diagnosis and therapeutic management.

Some large bone defects increase the risk of spontaneous fracture, a rare complication of PHPT. The reported crude fracture rate in patients with PHPT has been documented as 15 per 1000 person-years (compared with 8 per 1000 in controls) [32]. However, PHPT revealed by pathological fracture, as in our third patient, is extremely rare [24, 32].

Brown tumors exhibit no pathognomonic histological changes and are characterized by numerous giant cells, diffused or arranged in clusters, in a background of mononuclear oval-to-spindle stromal cells [27]. These giant cells are similar to those in other giant cell lesions such as reparative giant cell granulomas, cherubism, and aneurysmal bone cysts. Therefore, distinguishing a brown tumor from other giant cell lesions on the basis of histological examination may be difficult [33, 34]. A definitive diagnosis is only possible by comparing the clinical manifestations and radiological and laboratory test results that differentiate the lesions.

The treatment of choice for PHPT is parathyroidectomy, and the best treatment of brown tumors is cure of the underlying PHPT [3, 17, 18]. However, the fracture should undergo curettage, associated enucleation, and stabilization in patients with large lesions or lesions that persistently grow despite treatment, persist for more than 6 months, disrupt the function of the affected organ, or are associated with previous fracture fragility [35–38]. Extensive bone resection was not performed for our third patient because he had serious osteoporosis, making surgical intervention a high-risk procedure. In some cases, resection is performed only to achieve a definitive diagnosis [5, 28, 38].

In summary, PHPT is now usually detected in the early and asymptomatic phase because of recent improvements in analytical techniques. A brown tumor is a benign clinical entity appearing as an extremely rare manifestation of HPT and an uncommon cause of pathological fractures, which can be difficult to distinguish from other tumors, tumor-like lesions, and metastatic disease. When a patient, especially a middle-aged patient, presents with unexplained lytic bone lesions or pathological fractures, surgeons, endocrinologists, and especially radiologists should be reminded of this unusual presentation of PHPT to avoid unnecessary surgical removal. Our cases highlight the importance of a thorough diagnostic work-up for PHPT. A definitive diagnosis is only possible upon completion of clinical, radiological, and biochemical analysis.

### Consent

Written informed consent was obtained from the patients for publication of these case reports and any accompanying images. A copy of the written consent is available for review by the Editor of this journal. This study was approved by the ethics committee of the First Affiliated Hospital of Shantou University Medical College.
